# Photobiomodulation Therapy Combined with Static Magnetic Field (PBMT–SMF) on Spatiotemporal and Kinematics Gait Parameters in Post-Stroke: A Pilot Study

**DOI:** 10.3390/life12020186

**Published:** 2022-01-27

**Authors:** Arislander Jonathan Lopes Dumont, Heliodora Leão Casalechi, Shaiane Silva Tomazoni, Luanda Collange Grecco, Manuela Galli, Claudia Santos Oliveira, Ernesto Cesar Pinto Leal-Junior

**Affiliations:** 1 Laboratory of Phototherapy and Innovative Technologies in Health (LaPIT), Post-Graduated Program in Rehabilitation Sciences, U Nove de Julho University (UNINOVE), São Paulo 01525-000, Brazil; heliodoracasalechi@uni9.edu.br (H.L.C.); ernesto.leal.junior@uni9.pro.br (E.C.P.L.-J.); 2 Physiotherapy Research Group, Department of Global Public Health and Public Care, University of Bergen (UiB), 5020 Bergen, Norway; shaiane.tomazoni@eljconsultancy.com; 3 Vento Leste-Specialized Childcare Habilitation, Sorocaba 18046-715, Brazil; contato@clinicaventoleste.com.br; 4 Laboratory of Integrated Human Movements, University de Sorocaba, Sorocaba 18023-000, Brazil; 5 Center of Pediatric Neurostimulation, São Paulo 01229-000, Brazil; 6 Department of Electronic Information and Bioengineering, Politecnico di Milano, 20133 Milan, Italy; manuela.galli@polimi.it; 7 Health Sciences Program, Santa Casa de São Paulo School of Medical Sciences, São Paulo 01221-020, Brazil; claudia.oliveira@unievangelica.edu.br; 8 Postgraduate Program, University Center of Anapolis, Anápolis 75083-515, Brazil; 9 ELJ Consultancy-Scientific Consultants, São Paulo 04076-000, Brazil

**Keywords:** gait, low-level laser therapy, light-emitting diode therapy, physical therapy, photobiomodulation therapy, rehabilitation, stroke

## Abstract

Background: Gait deficit is a major complaint in patients after stroke, restricting certain activities of daily living. Photobiomodulation therapy combined with a static magnetic field (PBMT-SMF) has been studied for several diseases, and the two therapies are beneficia. However, their combination has not yet been evaluated in stroke. Therefore, for PBMT–SMF to be used more often and become an adjunctive tool in the rehabilitation of stroke survivors at physical therapy rehabilitation centers and clinics, some important aspects need to be clarified. Purpose: This study aimed to test different doses of PBMT–SMF, to identify the ideal dose to cause immediate effects on the spatiotemporal and kinematic variables of gait in post-stroke patients. Methods: A randomized, triple-blinded, placebo-controlled crossover pilot study was performed. A total of 10 individuals with hemiparesis within 6 months to 5 years since the occurrence of stroke, aged 45–60 years, were included in the study. Participants were randomly assigned and treated with a single PBMT–SMF dose (sham, 10 J, 30 J, or 50 J) on a single application, with one dose per stage at 7-day intervals between stages. PBMT–SMF was applied with a cluster of 12 diodes (4 of 905 nm laser, 4 of 875 nm LEDs, and 4 of 640 nm LEDs, SMF of 35 mT) at 17 sites on both lower limbs after baseline evaluation: plantar flexors (2), knee extensors (9), and flexors (6). The primary outcome was self-selected walking speed, and the secondary outcomes were kinematic parameters. Gait analysis was performed using SMART-D 140^®^ and SMART-D INTEGRATED WORKSTATION^®^. The outcomes were measured at the end of each stage after the single application of each PBMT–SMF dose tested. Results: No significant differences (*p* > 0.05) in spatiotemporal variables were observed between the different doses, compared with the baseline evaluation. However, differences (*p* < 0.05) were observed in the kinematic variable of the hip in the paretic and non-paretic limbs, specifically in the minimum flexion/extension angulation during the support phase (HMST–MIN) in doses 10 J, 30 J, and 50 J. Conclusions: A single application of PBMT–SMF at doses of 10 J, 30 J, and 50 J per site of the lower limbs did not demonstrate positive effects on the spatiotemporal variables, but it promoted immediate effects in the kinematic variables of the hip (maximum and minimum flexion/extension angulation during the support phase) in the paretic and non-paretic limbs in post-stroke people.

## 1. Introduction

Stroke is classified as a neurological deficit caused by an acute focal lesion of the central nervous system resulting from a vascular cause [1]. On a global scale, stroke ranks third in diseases with the greatest financial burden [2] and is one of the main causes of disability in adults [3]; stroke events are expected to increase dramatically [4]. Post-stroke sequelae are heterogeneous; however, after injury, people may experience sensorimotor changes, usually on one side of the body, such as hemiparesis, which results in muscle weakness, eventually leading to muscle spasticity and joint stiffness [3,4]. These changes directly influence the gait of post-stroke individuals, which consequently affects their level of activity, restricting their participation in the community and impacting their quality of life [5,6,7]. Hemiparetic gait is characterized mainly by the prolonged support of the non-paretic limb (support phase) and an increase in the swing phase of the paretic limb, resulting in a decrease in gait speed [8]. However, changes in spatiotemporal variables contribute to the poor kinematic performance of gait [9], which leads to higher metabolic cost [10] and insufficiency [11].

Photobiomodulation therapy (PBMT) using low-level laser and/or light-emitting diodes (LEDs) involves the administration of light at an intensity of 1–500 mW, which has no thermal or ablative effects [12]. The effects of PBMT are photochemical and photophysical, i.e., absorbed light causes a chemical change in the tissues [12]. The isolated effects of static magnetic field (SMF) are still unclear, but studies report that the use of SMF results in effects such as decreased oxidative stress, increased antioxidant activity, and increased production of adenosine triphosphate (ATP) [13,14,15]. However, the combination of PBMT and SMF (PBMT–SMF) demonstrated remarkable synergy, leading to enhanced electron transfer and consequent activation of the mitochondrial respiratory chain and ATP production [16]. In addition, studies have shown that PBMT–SMF improves muscle performance in healthy individuals [17,18] and athletes [19,20], decreases pain intensity in people undergoing total hip arthroplasty [21], decreases dyspnea intensity in people with chronic obstructive pulmonary disease [22], and improves functional mobility in post-stroke people [23].

Improving the quality of gait and walking safety is one of the main objectives of the management of post-stroke people [24]. In this context, there are several techniques for gait rehabilitation for these patients, such as aerobic training, functional electrical stimulation, multidimensional rehabilitation, robotics, sensory stimulation training, strength/resistance training, task-specific locomotor rehabilitation, and visually guided training [25]. Thus, physical therapy is described as one of the most used and highly successful techniques in gait rehabilitation of post-stroke patients [26]. However, promising resources have emerged as new tools in post-stroke rehabilitation, including PBMT–SMF [23]. The positive effects of PBMT–SMF with a dose of 30 J were demonstrated in post-stroke patients; however, these effects were observed in variables related to functional mobility [23]. In the mentioned study [23], these effects were measured from the tests: six-minute walk test (6 MWT) and timed up and go (TUG). It is known that the effects of PBMT–SMF are dose dependent, and this has been demonstrated for different variables in several clinical conditions, but these effects have not yet been clearly demonstrated for the kinematic parameters evaluated in the present study. Therefore, for PBMT–SMF to be used more often and become an adjunctive tool in the rehabilitation of stroke survivors at physical therapy rehabilitation centers and clinics, some important aspects need to be clarified, particularly with regard to the ideal dose and other parameters to be used for this population.

Therefore, the aim of this study was to test different doses of PBMT–SMF, to identify the ideal dose to cause immediate effects on the spatiotemporal and kinematic variables of gait in post-stroke people.

## 2. Materials and Methods

### 2.1. Study Design

A randomized, triple-blinded (assessor, therapists, and participants), sham-controlled, crossover pilot study was conducted in accordance with the Declaration of Helsinki and the guidelines for research involving human subjects. This study was approved by the Human Research Ethics Committee of University Nove de Julho (certificate number: 1.463.512) and registered at ClinicalTrials.gov (accessed on 1 October 2021) (NCT03653299). We recruited a convenience sample of 10 post-stroke patients, based on a sample used in a previous dose–response study using the same device [23]. Since the study has a crossover design, this represents the total number of individuals (*n* = 10). To compensate for a possible 20% dropout rate, 12 people were eventually recruited. All participants received full information regarding the objectives of the study and procedures to be performed, and they signed a statement of informed consent. Moreover, the patients were informed that they could drop out of the study at any time with no negative consequences. The study was performed in the Laboratory of Phototherapy and Innovative Technologies in Health (LaPIT), University Nove de Julho, São Paulo-SP, Brazil, in four stages with seven-day intervals between stages. 

### 2.2. Participants

People with a medical diagnosis of a single ischemic or hemorrhagic stroke 6 months to 5 years after the occurrence of stroke and who met the eligibility criteria were included. People of any sex, aged 45–60 years, with hemiparesis from a single stroke event, with crural predominance occurring within the time frame, and receiving conventional standardized physical therapy at university clinics were included. Other inclusion criteria were the ability to walk barefoot with or without a gait-assistive device, controlled and clinically stable comorbid diseases, the capacity to read and understand people’s information charts, or the capacity to sign an informed consent statement. Further, people with fixed deformities of the lower limbs, treatments with botulinum toxin and/or neurolytic blocks in the previous 6 months, a history of osteoarticular disorders, any other health condition that would affect gait performance, cognitive deficits that would affect test performance, those who had undergone surgery, and those who did not meet the inclusion criteria were excluded from the study.

### 2.3. Blinding 

All clinical assessments were conducted by an assessor who was blinded to treatment allocation. Neither the therapists nor participants were aware of whether a sham or active treatment was being administered. The same PBMT–SMF device was used for all irradiated doses and the sham. To ensure blinding for therapists and participants, the PBMT–SMF device emitted the same sounds and displayed the same information regardless of the programmed dose or mode. Finally, the researcher who evaluated the outcomes and the researcher who performed the data analysis were not aware of their irradiated dose order before the end of the study. Only the researcher in charge of the randomization process and programming of the PBMT–SMF device had the identifying code to determine which treatment should be administered. This researcher was instructed not to disclose the PBMT–SMF dose to any of the patients or other researchers involved until the end of the study.

### 2.4. Randomization 

The people received 4 weeks of PBMT–SMF, with a different dose applied each week (sham, 10 J, 30 J, and 50 J per site). The treatment order was randomized. We generated codes through the random.org website to ensure that at stage 1, an equal number of people received sham, 10 J, 30 J, and 50 J doses, respectively. The other stages (2, 3, and 4) also incorporated 25% of the people per dose, in order to counterbalance the number of people tested between the doses (sham, 10 J, 30 J, and 50 J per site) during the four stages (one dose each stage/week). All people started and finished the treatment at the same time. The randomization was balanced (3:2:2:3) to ensure the distribution of doses according to the stage. In the first session, each patient was allocated according to the randomization codes (A, B, C, and D) that determined the sequence of doses to be administered in each stage. Over the four stages, patients received different doses of PBMT–SMF each week according to the four different sequences: A (sham, 10 J, 30 J, and 50 J), B (10 J, 30 J, 50 J, and sham), C (30 J, 50 J, sham, and 10 J), and D (50 J, sham, 10 J, and 30 J). Allocation concealment was achieved using sequentially numbered, sealed, and opaque envelopes.

### 2.5. Outcomes Measurements

The primary outcome was self-selected walking speed, and the secondary outcomes were the other spatiotemporal variables, in addition to the kinematic variables of gait. Evaluations were performed at baseline and after a single application of each PBMT–SMF dose tested (sham, 10 J, 30 J, and 50 J). A member of the research team who did not interact with people during the interventions or evaluations exported the data to spreadsheets and sent the data to the statistician.

Gait analysis was performed with SMART-D 140^®^ (BTS Engineering-BTS, Milan-ITA) with a sampling rate of 100 Hz, involving the use of eight cameras sensitive to the infrared spectrum and SMART-D INTEGRATED WORKSTATION^®^ with 32 analog channels. All people wore swimsuits to facilitate the placement of reflective markers. After anthropometric measurements (height, weight, lower limb length, distance between the femoral condyles or diameter of the knee, distance between the malleoli or diameter of the ankle, and distance between the anterior superior iliac spine and thickness of the pelvis—the vertical distance on the sagittal plane of the supine subject between the anterior superior iliac spine and great trochanter), passive markers were placed at specific reference points directly on the skin to evaluate the kinematics of each segment of the body, as described in the literature [27]. After placing reflective markers, people were instructed to walk along a fixed 10 m walkway, the assessor gave the following voice command: “please, walk at a comfortable pace”; at least six attempts were made (three rounds and three rounds). For each participant, three out of the six trials that were consistent in terms of gait patterns were considered for analysis. All data were exported in .txt format to electronic spreadsheets and tabulated using Microsoft Office Excel 2013.

The following spatiotemporal variables were taken into consideration: velocity (m/s), (mean velocity of progression); step length (m, which is the longitudinal distance between the point of initial contact of one foot and the point of initial contact of the contralateral foot); step width (m, which is the distance between the rear end of the right and left heel centerlines along the mediolateral axis); stance phase (% gait cycle, which is the% of gait cycle that begins with the initial contact and ends with toe-off of the same limb); double support (s, which is the period of time when both feet are in contact with the ground).

The following kinematic variables were taken into consideration (degrees): pelvis, PT–IC = angle of pelvic tilt at initial contact; PT–MAX = maximum angle of pelvic tilt; PT–MIN = minimum angle of pelvic tilt; PT–ROM = range of motion of pelvic tilt; PO–MAX = maximum angle of pelvic obliquity; PO–MIN = minimum angle of pelvic obliquity; PO–ROM = range of motion of pelvic obliquity; PR–MAX = maximum angle of pelvic rotation; PR–MIN = minimum angle of pelvic rotation; PR–ROM = range of motion of pelvic rotation. Hip, HIC = angle of hip flexion at initial contact; HMST–MAX = maximum angle of hip flexion/extension in stance; HMST–MIN = minimum angle of hip flexion/extension in stance; HMST–ROM = range of motion of hip flexion/extension in stance; HAA–MAX = maximum angle of hip abduction/adduction; HAA min = minimum angle of hip abduction/adduction; HAA–ROM = range of motion of hip abduction/adduction; HROT–IC = range of motion of hip rotation at initial contact; HROT–MEAN = mean value of hip rotation. Range of motion was computed as the difference between the maximum and minimum values of the specific plot. Knee, KIC = angle of knee flexion at initial contact; KMSW = maximum angle of knee flexion in swing; KMST = minimum angle of knee flexion in stance; K–ROM = range of motion of the knee on the sagittal plane; the range of motion was computed as the difference between the maximum and minimum (KMST index) value of the plot. Ankle, AIC = angle of ankle dorsiflexion/plantar flexion at initial contact; AMST–MAX = maximum angle of ankle dorsiflexion in stance; AMST–MIN = minimum angle of ankle plantar flexion in stance; AMSW = maximum angle of ankle dorsiflexion in swing; A–ROMST = range of motion of the ankle joint during stance phase was computed as the difference between the maximum and minimum (AMST index) value of the plot. Foot, FP IC = foot progression angle at initial contact; FP MEAN = mean value of foot progression. All kinematic graphs obtained during the gait analysis were normalized as the percentage of the gait cycle, producing sagittal kinematic plots of the pelvis, hip, knee, and ankle for each cycle. The BTS Smart-D Clinic software (BTS, Italy) was used, with the data exported to .txt files.

### 2.6. Intervention

**Figure 1:**
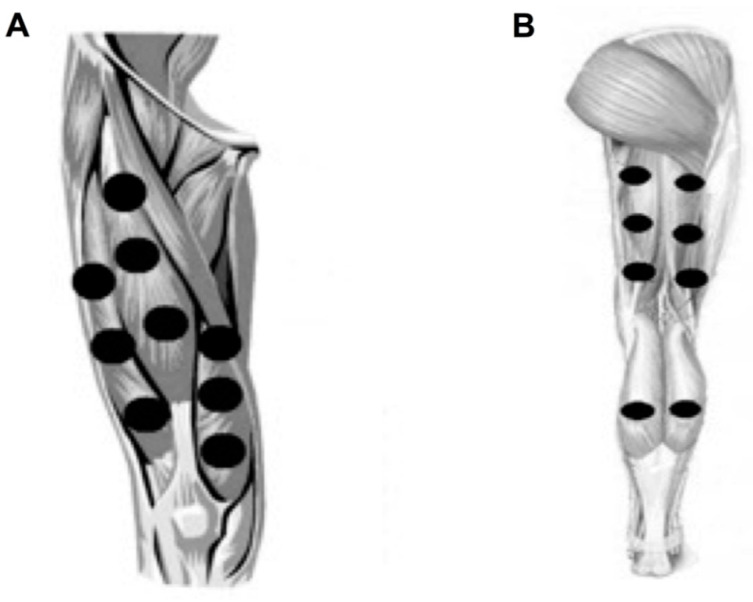
(**A**) Sites of application of PBMT+ SMF to the knee extensors muscles at both lower limbs; (**B**) sites of application of PBMT + SMF to the knee flexors muscles and plantar flexor muscles at both lower limbs.

A single application of different doses of PBMT–SMF was administered after the baseline pre-intervention evaluation. In each stage, the patients received different doses of PBMT–SMF according to previous randomization (sham, 10 J, 30 J, and 50 J); at the end of the stage, the primary and secondary outcomes were assessed. A washout of one week was performed between one dose and another, and this washout was one week. Recent studies [23,28] determined that the technology of the equipment we used in the present study demonstrated that the ergogenic effects do not last longer than 54 h after irradiation; therefore, the 7-day washout used in the present study was adequate. PBMT–SMF was administered in direct contact with the skin and applied with slight pressure at nine sites on the knee extensors (Figure 1A), six sites on the knee flexors, and two sites on the plantar flexor muscles (Figure 1B), on both lower limbs.

PBMT–SMF was administered using a device that delivered PBMT and SMF simultaneously on the same device. PBMT–SMF was administered using a cluster of 12 diodes: 4 laser diodes of 905 nm (mean power of 0.3125 mW and peak power of 12.5 W for each diode), 4 LED diodes of 875 nm (mean power of 17.5 mW for each diode), 4 LED diodes of 640 nm (mean power of 15 mW for each diode), and an SMT of 35 mT. The device was manufactured by Multi Radiance Medical^®^ (Solon, OH, USA). The cluster used in this study was circular and had an area of 20 cm^2^. Based on the randomization schedule, the people received PBMT–SMF at the following doses: 10 J per area (76 s of irradiation in each site), 30 J per area (228 s of irradiation in each site), 50 J per area (380 s of irradiation in each site), or sham (152 s of placebo irradiation in each area and no effective irradiation). The sham irradiation was identical to the actives, and the device displayed the same settings and emitted the same sound regardless of the dose (even for the placebo). Table 1 provides a full description of PBMT–SMF.

### 2.7. Data Analysis

Intention-to-treat analysis was performed a priori. The Shapiro–Wilk test was used to verify the normal distribution of data. Parametric data and data from the analysis of spatiotemporal and kinematic gait variables were expressed as mean and standard deviation (SD). Repeated measures ANOVA with intra-patient data and Bonferroni post hoc test were used for comparisons. Statistical analysis was performed using SPSS (v.19.0), with the level of significance set at 5% (*p* < 0.05). Data are expressed as mean (±SD) in the tables and as mean (±SEM) in graphs to allow better presentation of data.

## 3. Results

**Figure 2:**
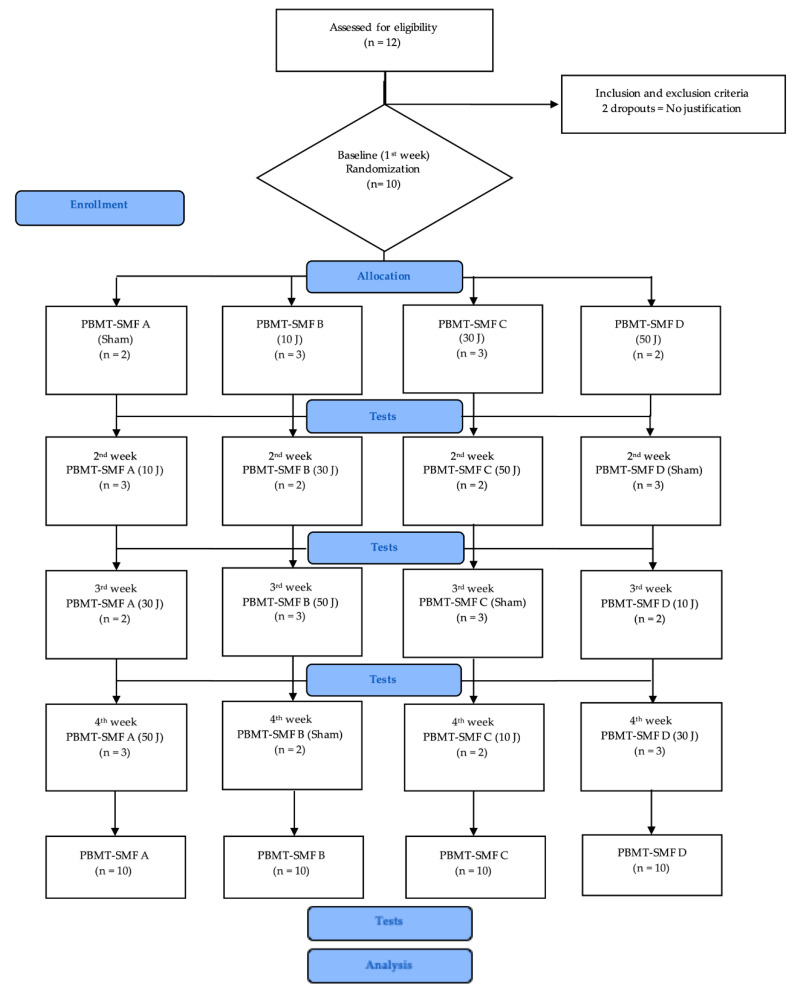
Study flowchart in accordance with the CONSORT statement. Legend: J = joule; *n* = number of patients per group; PBMT = photobiomodulation therapy; SMF = static magnetic fields.

Twelve people were initially recruited; however, two dropped out without explaining their reasons before randomization. Thus, 10 people were randomized and analyzed for each treatment dose sequence. All procedures of the study adhere to the CONSORT guidelines and are summarized in a flowchart (Figure 2). All people received a treatment dose according to randomization. The baseline characteristics of the people are summarized in Table 2.

The results of the spatiotemporal gait variables under the different conditions tested (sham, 10 J, 30 J, and 50 J) are summarized in Table 3. Statistical analysis showed no significant differences (*p* > 0.05) in the comparative analysis of treatment doses, compared with both the baseline and between the treatment doses.

**Figure 3:**
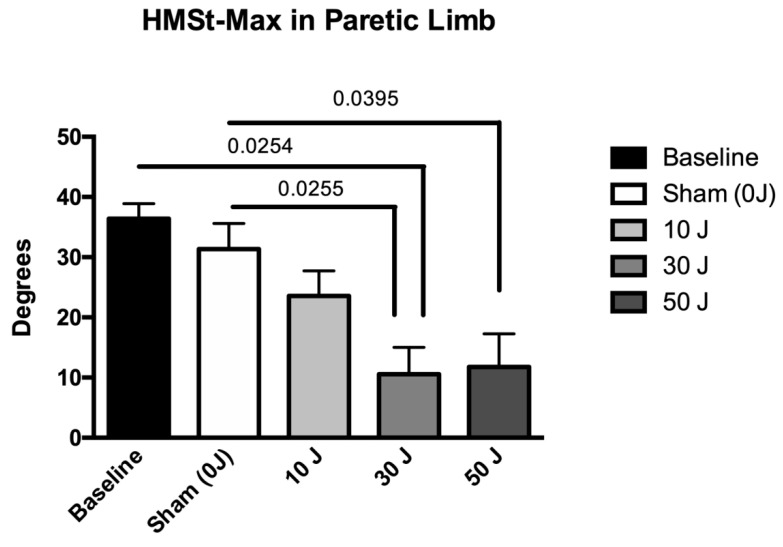
The results in the variable maximum angle of hip flexion/extension in stance (HMST–MAX) in the paretic limb (data expressed as mean ± SEM). Legend: HMST–MAX = maximum angle of hip flexion/extension in stance.

**Figure 4:**
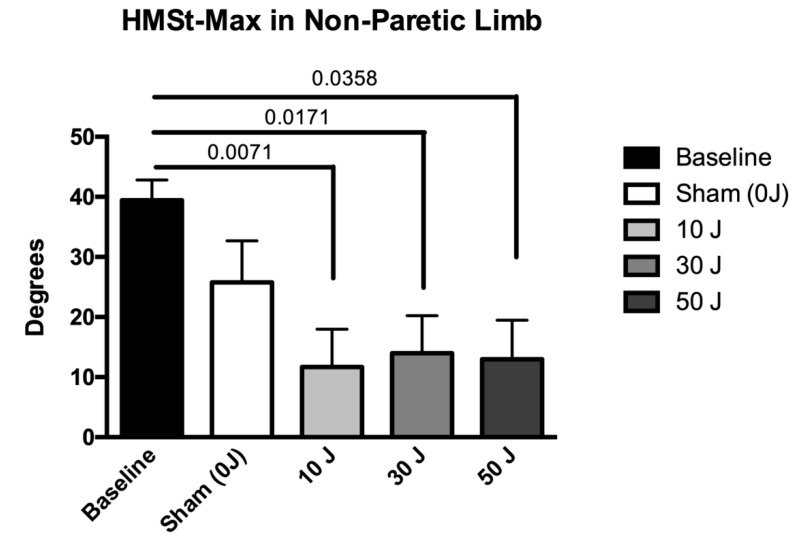
The results in the variable maximum angle of hip flexion/extension in stance (HMST–MAX) in the non-paretic limb (data expressed as mean ± SEM). Legend: HMST–MAX = maximum angle of hip flexion/extension in stance.

**Figure 5:**
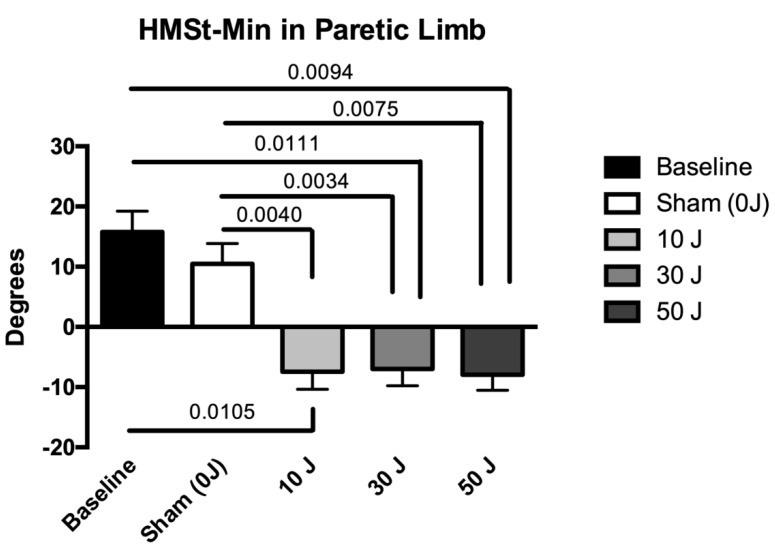
The results in the variable minimum angle of hip flexion/extension in stance (HMST–MIN) in paretic limb (data expressed as mean ± SEM). Legend: HMST–MIN = minimum angle of hip flexion/extension in stance.

**Figure 6:**
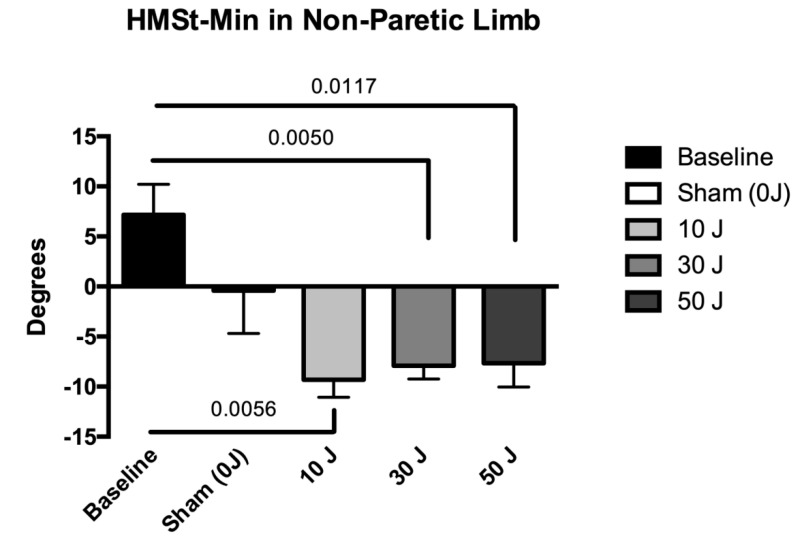
The results in the variable minimum angle of hip flexion/extension in stance (HMST–MIN) in non-paretic limb (data expressed as mean ± SEM). Legend: HMST–MIN = minimum angle of hip flexion/extension in stance.

The results of kinematic gait variables under different conditions are summarized in Table 4. The dose of 30 J showed statistically significant improvements in the variable HMST–MAX in the paretic limb (*p* < 0.05), compared with the baseline value, and the doses of 30 J and 50 J showed statistically significant improvements in the variable HMST–MAX in the paretic limb (*p* < 0.05), compared with sham (Figure 3). In the non-paretic limb, statistically significant differences were also found in the variable HMST–MAX at doses of 10 J, 30 J, and 50 J (*p* < 0.05), compared with the baseline values (Figure 4). The 10 J, 30 J, and 50 J doses showed statistically significant improvements in the variable HMST–MIN in the paretic limb (*p* < 0.05), compared with the baseline value and sham (Figure 5). In the non-paretic limb, statistically significant differences were also found in the variable HMST–MIN at doses of 10 J, 30 J, and 50 J (*p* < 0.05), compared with the baseline value (Figure 6). The other variables of kinematic gait showed no significant differences (*p* > 0.05) in the comparative analysis of treatment doses, compared with baseline (Table 4). None of the patients reported any adverse events.

## 4. Discussion

To our knowledge, this is the first study that tests different doses of PBMT–SMF to identify the ideal dose to cause immediate effects on the spatiotemporal and kinematic variables of gait in post-stroke people.

The statistical analysis showed no significant differences in spatiotemporal variables (self-selected walking speed, step width, step length, support, and double support) between the different doses tested (sham, 10 J, 30 J, and 50 J), compared with the baseline values. In contrast, statistically significant differences were observed in the kinematic variables of the hip in the paretic and non-paretic limbs, specifically in the maximum and minimum angle of hip flexion/extension in stance. In the paretic limb, the 30 J dose was able to decrease the maximum angle of hip flexion/extension in stance, compared with the baseline value, and the 30 J and 50 J doses decreased the maximum angle of hip flexion/extension in stance, compared with sham, whereas the non-paretic limb demonstrated a decrease in maximum angle of hip flexion/extension in stance in relation to the baseline value with the 10 J, 30 J, and 50 J doses. In relation to the minimum angle of hip flexion/extension in stance, in the paretic limb, the 10 J, 30 J, and 50 J doses also demonstrated a decrease in relation to the baseline value and sham. In the non-paretic limb, the 10 J, 30 J, and 50 J doses also demonstrated favorable effects on the decrease in minimum angle of hip flexion/extension in stance of the non-paretic limb, compared with the baseline value and sham.

The functional mobility of post-stroke people is directly affected by poor gait performance, which restricts certain activities of daily living and consequently affects the quality of life [29]. Thus, gait rehabilitation plays an extremely important role in the rehabilitation of post-stroke people. Different techniques of physical therapy for gait rehabilitation in post-stroke people have been described [30]. However, the physiological and biomechanical mechanisms of several interventions in the improvement of spatiotemporal and kinematic variables of gait are not well understood [25,31]. Many studies on interventions for post-stroke locomotor rehabilitation have only assessed changes in functional recovery [25,31]. For example, a previous trial used the same model of PBMT device as our trial with a dose of 30 J per site and observed positive effects of PBMT–SMF on the functional mobility of post-stroke people [23]. The positive results shown in the functional mobility of these people justify the importance to investigate the effects of PBMT–SMF on spatiotemporal and kinematic variables of gait, since most of the studies evaluate functional mobility.

Gait speed is a complex functional activity, a type of multimodal product of many processes [31]. One of the hypotheses for obtaining a significant improvement in gait speed in post-stroke people is the restoration of the range of motion of the joints during the gait cycle, that is, the improvement in the kinematic variables of gait [25]. Among the spatiotemporal variables of post-stroke people, gait speed proved to be a predictor of independence in terms of functional disability and quality of life [32]. However, our study evaluated the effect of PBMT–SMF on gait speed and did not find positive effects for any of the doses of PBMT–SMF used. Regarding the other spatiotemporal variables (step width, length step, support, and double support), positive effects were also not observed, and rehabilitation techniques that demonstrate potential in the recovery of these variables are the techniques used in a chronic way, that is, in repetitive sessions such as gait training [24]. This may be related to the lack of positive results observed in the present study, which verified only the acute effects of PBMT–SMF.

Chronic post-stroke people assume a non-pathologic pattern of walking and compensatory strategies that alter the whole gait kinematics. This is due to the lack of motor control and the presence of muscle weakness and muscle spasticity of the paretic limb [5]. A strategy often used by post-stroke people to walk is the prolonged support of the non-paretic limb, surrounding the paretic limb, using the trunk swing in a compensatory way to move the paretic limb, resulting in insufficient flexion and extension of both hips [32,33,34,35,36]. Moreover, when the paretic limb is in the support phase, knee hyperextension occurs [37,38] due to the spasticity of the extensor musculature and the weakness of the knee flexor muscles [5]. There is also insufficient plantar dorsiflexion of the paretic limb, making it difficult to propel the limb into a new gait cycle [33]. According to the results of the present study, a single application of PBMT–SMF at different points of the lower limbs was able to promote improvements in the minimum flexion/extension of the hip of both limbs. These data are relevant, once that the good performance in flexion and extension of the hip is of paramount importance for gait in healthy people [39] and hip flexion/extension is one of the main kinematic changes in gait in post-stroke people [5,33,34,35]. In addition, the values obtained in our study for minimum flexion/extension of the hip in post-stroke patients demonstrate that this variable is closer to the values found for healthy people [39], highlighting the importance of these results. However, the values obtained in our study for maximum hip flexion/extension in post-stroke people demonstrate that this variable is more out of physiological values for healthy people [39]. We believe that the values obtained in the variable maximum hip flexion/extension in the present study either compensated for the change obtained in the minimum hip flexion/extension.

Thus, our findings suggest that PBMT–SMF at doses of 10 J, 30 J, and 50 J promote acute changes in the hip flexor and extensor muscles in both lower limbs (non-paretic and paretic limbs), consequently causing improvement in hip mobility. Even without muscle function evaluation, these results agree with previous studies that demonstrated the positive effects of PBMT–SMF on muscle performance in healthy individuals and athletes [17,19,20]. The satisfactory results of our trial can also be attributed to the choice of parameters, which followed the clinical and scientific recommendations of PBMT and PBMT–SMF for large muscle groups of healthy individuals [13,40].

Isolated effects of PBMT have been described in studies that evaluated the muscular function of the paretic limb in post-stroke people. It has been shown that a single application of PBMT at 30 points distributed in the muscles of the paretic limb of stroke people can increase muscle performance and significantly decrease blood lactate levels [41]. In addition, another study using PBMT at 30 points distributed along the musculature of the paretic limb observed an increase in the time of onset of muscle fatigue [42]. Although we did not observe changes in relation to the acute effects of PBMT–SMF in spatiotemporal gait variables, the results of the kinematic variables are of great value, since in the clinical scenario, restoring kinematics is the first step for the rehabilitation of gait in post-stroke people [25].

Further studies are important to assess muscle activity, along with a three-dimensional gait assessment after the application of PBMT–SMF, once the non-sagittal movements were determinant for the gait mechanics in strokes [43]. In addition, further studies may consider the application of PBMT–SMF protocols associated with some type of motor therapy [44] in post-stroke people. The limitation of the present study was that 12 people were initially recruited, but 2 dropped out prior to randomization without explaining their reasons. In addition, the sample size was calculated for the large study (which contained several outcomes), and even though it was a crossover study, the number of people included in the study was small. The lack of tools for evaluating muscle activity is also a limitation.

## 5. Conclusions

The application of PBMT–SMF at doses of 10 J, 30 J, and 50 J per site of the lower limbs did not show positive effects on spatiotemporal gait variables in post-stroke people. However, the same doses had positive effects on kinematic variables maximum and minimum angle of hip flexion/extension in stance.

## Figures and Tables

**Table 1:** PBMT–SMF parameters.

Parameters	Treatment with 10 J, 30 J and 50 J
Number of Lasers	4 Super-Pulsed Infrared
Wavelength (nm)	905 (±1)
Frequency (Hz)	250
Peak power (W)-each	12.5
Average mean optical output (mW)-each	0.3125
Power density (mW/cm^2^)-each	0.71
Dose/Energy density (J/cm^2^)-each	0.054, 0.162, 0.271
Energy (J)-each	0.02375, 0.07125 or 0.11906
Spot size of laser (cm^2^)-each	0.44
Number of red LEDs	4 Red
Wavelength of red LEDs (nm)	640 (±10)
Frequency (Hz)	2
Average optical output (mW)-each	15
Power density (mW/cm^2^)-each	16.67
Dose/Energy density (J/cm^2^)-each	1.27, 3.8 and 6.35
Energy (J)-each	1.14, 3.42 or 5.72
Spot size of red LED (cm^2^)-each	0.9
Number of infrared LEDs	4 Infrared
Wavelength of infrared LEDs (nm)	875 (±10)
Frequency (Hz)	16
Average optical output (mW)-each	17.5
Power density (mW/cm^2^)-each	19.44
Dose/Energy density (J/cm^2^)-each	1.48, 4.43 or 7.41
Energy (J)-each	1.33, 3.99 or 6.67
Spot size of LED (cm^2^)-each	0.9
Number of magnets	1
Shape	Ring
Area (cm^2^)	20
Width (cm)	0.5
Thick (cm)	2
Magnetic field (mT)	35
Irradiation time per site (sec)	76, 228, or 381
Total energy per site (J)	10, 30 or 50
Total energy applied per lower limb (J)	170, 510 or 850
Aperture of device (cm^2^)	20
Application mode	Cluster probe held stationary in skin contact with a 90-degree angle and slight pressure

Legend: (cm^2^) = square centimeter; (cm) = centimeter; (mT) = millitesla; (mW) = megawatt; (nm) = nanometer; (sec) = seconds; (W) = watt; (Wz) = hertz; LED = light-emitting diodes.

**Table 2:** Anthropometrical characteristics of samples (data expressed as mean (±SD) and absolute frequency).

Individuals (*n*)	10
Age (years)	58.5 (±10.04)
Body mass (kg)	72.3(±13.8)
Height (m)	1.69 (±0.10)
BMI (kg/m^2^)	27.3(±6.8)
Time since stroke (months)	42.2 (±19.4)
Male/Female	6/4
Type of stroke (ischemic/hemorrhagic)	5/5
Main stroke lesion (cortical/subcortical)	6/4
Affected side (right/left)	4/6
Gait-assistance device (cane/braces)	6/1

Legend: mean ± SD, (*n*) = number, (kg) = kilogram, (m) = meter, (kg/m^2^) = kilogram/square meters.

**Table 3:** Outcomes of spatiotemporal gait variables, expressed as mean and standard deviation (±SD).

.			DOSE				
			BASELINE *n*= 10	SHAM *n* = 10	10 J *n* = 10	30 J *n* = 10	50 J *n* = 10
**EVALUATION**		**VELOCITY** **(m/s)**	0.406 (0.143)	0.418 (0.127)	0.41 (0.092)	0.497 (0.135)	0.393 (0.089)
		**STEP WIDTH** **(m)**	0.342 (0.277)	0.221 (0.041)	0.219 (0.039)	0.227 (0.044)	0.230 (0.047)
	**NON-PARETIC**	**STANCE PHASE** **(%GC)**	67.148 (5.239)	72.734 (4.058)	71.426 (5.941)	73.793 (5.783)	72.976 (4.586)
		**DOUBLE SUPPORT** **(%GC)**	15.091 (4.543)	18.667 (4.040)	22.386 (9.628)	21.797 (8.569)	17.822 (4.073)
		**STEP LENGTH** **(m)**	0.355 (0.079)	0.342 (0.096)	0.324 (0.096)	0.332 (0.114)	0.341 (0.103)
	**PARETIC**	**STANCE PHASE** **(%GC)**	61.010 (6.334)	65.347 (4.530)	65.6 (4.179)	63.050 (4.981)	63.652 (4.408)
		**DOUBLE SUPPORT** **(%GC)**	13.076 (1.745)	19.69 (5.489)	15.692 (3.364)	15.941 (3.429)	18.510 (4.351)
		**STEP LENGTH** **(m)**	0.380 (0.093)	0.323 (0.123)	0.304 (0.110)	0.317 (0.137)	0.299 (0.112)

Legend: (m) = meters; (m/s) = meters per second; (% GC) = percentage of gait cycle.

**Table 4:** Outcomes of kinematics gait variables, expressed as mean and standard deviation (±SD).

		NON-PARETIC					PARETIC				
EVALUATION		BASELINE	SHAM	10 J	30 J	50 J	BASELINE	SHAM	10 J	30 J	50 J
**PELVIS**	**PT–IC**	11.603(6.522)	12.662 (6.49)	9.716 (5.231)	9.343 (5.52)	11.141 (5.99)	8.11 (6.350)	9.983 (6.065)	7.79 (5.682)	6.645 (5.87)	8.26 (4.882)
	**PT–MAX**	14.425 (6.78)	16.11 (6.58)	12.951 (6.665)	12.193 (5.77)	13.88 (6.290)	14.843 (6.661)	16.01 (6.751)	12.985 (6.801)	12.063 (5.97)	14.33 (6.12)
	**PT–MIN**	6.283 (5.911)	8.033 (5.800)	5.321 (4.89)	4.59 (4.61)	6.31 (3.551)	6.073 (5.57)	8.255 (6.13)	6.29 (5.02)	4.45 (4.571)	6.18 (3.72)
	**PT–ROM**	8.333 (3.474)	8.055 (4.99)	7.63 (3.770)	7.913 (5.051)	7.381 (4.90)	8.513 (4.317)	7.751 (5.195)	7.70 (3.624)	8.013 (4.935)	8.03 (5.202)
	**PO–MAX**	3.173 (5.40)	8.70 (9.10)	10.863 (8.071)	12.51 (10.314)	11.60 (8.815)	5.04 (4.145)	5.174 (5.195)	2.093 (8.90)	3.233 (8.290)	2.18 (9.304)
	**PO–MIN**	−5.133 (3.68)	−6.39 (9.773)	−3.211 (7.133)	−2.76 (6.88)	−2.80 (9.354)	−3.13 (6.467)	−7.603 (7.80)	−11. 585 (8.64)	−11.47 (10.226)	−11.83 (8.585)
	**PO–ROM**	8.413 (4.142)	15.60 (8.064)	14.35 (6.604)	13.233 (6.393)	14.4 (7.093)	8.20 (4.88)	13.77 (4.694)	14.613 (6.820)	13.571 (5.904)	13.805 (6.902)
	**PR–MAX**	9.42 (9.303)	12.514 (6.713)	12.545 (7.375)	11.66 (7.571)	12.995 (7.111)	3.303 (10.83)	14.57 (6.804)	11.53 (6.28)	10.91 (5.862)	13.85 (6.131)
	**PR–MIN**	−3.445 (11.24)	0.74 (8.363)	0.213 (12.722)	−0.27 (12.442)	−0.418 (10.90)	−9.27 (10.905)	3.482 (9.89)	−0.60 (6.574)	−0.363 (8.05)	2.643 (8.23)
	**PR–ROM**	12.763 (7.560)	11.992 (7.84)	12.415 (9.15)	12.231 (9.58)	13.028 (10.51)	12.48 (5.79)	11.744 (6.343)	11.783 (5.09)	11.27 (5.76)	11.205 (6.433)
**HIP**	**HIC**	29.067 (15.60)	11.338 (27.45)	15.863 (23.864)	14.915 (23.94)	12.84 (25.563)	28.055 (18.38)	21.43 (26.81)	11.983 (21.45)	10.64 (20.79)	11.205 (22.70)
	**HMST–MAX**	39.45 (10.65)	25.77 (21.86)	11.69 (19.85) *	13.99 (19.69) *	12.99 (20.49) *	36.39 (7.856)	31.34 (13.45)	23.57 (13.1)	10.57 (14.11) *, #	11.78 (17.37) #
	**HMST–MIN**	7.174 (9.645)	−0.4053 (13.52)	−9.31 (5.533) *	−7.924 (4.163) *	−7.668 (7.494) *	15.77 (10.95)	10.47 (10.66)	−7. 41 (9.29) *, #	−6.971 (8.81) *, #	−7.924 (8.22) *, #
	**HMST–ROM**	29.85 (8.79)	25.011 (18.461)	25.13 (18.262)	22.42 (18.074)	21.62(15.902)	19.291 (9.252)	18.214 (9.90)	19.25 (11.79)	18.57 (11.56)	20.52 (14.744)
	**HAA–MAX**	7.15 (10.363)	12.462 (14.714)	8.355 (15.323)	10.10 (16.620)	7,25 (11.901)	4.30 (7.85)	8.07 (9.852)	12.17 (13.421)	12.833 (15.950)	8.34 (11.400)
	**HAA–MIN**	−0.65 (13.365)	−4.203 (10.052)	−2.543 (16.60)	−1.91 (12.10)	−2.623 (11.424)	−4.013 (5.675)	−3.59 (11.47)	1.843 (13.201)	2.66 (15.051)	−0.155 (10.122)
	**HAA–ROM**	9.00 (3.96)	13.95 (6.330)	11.415 (4.29)	10.88 (5.89)	9.8 (5.970)	8.42 (5.675)	9.38 (5.313)	11.38 (7.893)	10.183 (3.09)	9.65 (3.39)
	**HROT–IC**	8.883 (29.09)	17.07 (20.940)	12.043 (17.79)	15.97 (16.98)	19.925 (13.78)	17.81 (27.062)	13.65 (29.17)	12.805 (18.0)	8.601 (23.955)	14.36 (20.01)
	**HROT–MEAN**	12.771 (27.30)	8.671 (25.573)	5.784 (15.001)	10.59 (16.98)	14.333 (12.45)	21.118 (30.285)	12.303 (30.19)	9.84 (14.450)	5.860 (21.39)	9.76 (16.110)
**KNEE**	**KIC**	16.21 (13.99)	9.54 (13.91)	11.69 (16.012)	17.91 (13.84)	12.525 (11.493)	16.21 (13.999)	16.16 (16.585)	14.375 (15.354)	15.74 (13.681)	14.985 (11.53)
	**KMSW**	30.86 (13.51)	27.19 (23.353)	30.31 (22.89)	32.453 (21.481)	31.695 (21.632)	30.863 (13.505)	33.06 (13.993)	31.64 (16.07)	31.053 (12.305)	31.88 (12.28)
	**KMST**	13.40 (11.62)	5.881 (10.415)	6.43 (8.484)	5.453 (8.181)	4.641 (7.65)	12.40 (11.63)	8.65 (11.192)	6.352 (6.80)	5.79 (8.814)	8.155 (8.64)
	**K–ROM**	15.28 (11.88)	30.744 (20.392)	30.31 (22.892)	27.81 (20.121)	26.631 (19.022)	15.28 (11.885)	21.233 (15.56)	22.031 (14.623)	19.89 (14.223)	24.34 (15.944)
**ANKLE AND FOOT**	**AIC**	−1.97 (6.13)	4.99 (13.404)	1.463 (5.313)	2.59 (9.23)	0.76 (9.182)	−3.96 (8.66)	−0.629 (7.64)	−0.185 (5.332)	−1.76 (6.04)	−0.401 (6.921)
	**AMST–MAX**	12.29 (9.29)	19.3 (11.61)	16.975 (3.98)	16.68 (6.355)	15.331 (5.17)	9.11 (13.93)	13.714 (9.88)	13.325 (8.733)	12.93 (9.54)	13.001 (8.38)
	**AMST–MIN**	−5.15 (7.45)	−0.833 (14.017)	−1.185 (6.225)	−2.341 (8.75)	−4.882 (8.541)	−3.87 (10.51)	−0.6 (8.29)	−0.54 (5.40)	−2.45 (7.071)	−1.24 (6.404)
	**AMSW**	7.31 (9.69)	13.77 (13.17)	11.743 (8.531)	12.943 (9.018)	9.44 (9.37)	3.79 (12.43)	6.30 (7.88)	6.23 (6.22)	4.28 (6.572)	5.11 (7.235)
	**A–ROMST**	17.78 (9.54)	20.055 (8.72)	18.12 (7.115)	19.501 (9.404)	20.403 (7.565)	12.963 (6.79)	14.825 (7.551)	13.693 (6.49)	15.135 (6.41)	14.17 (6.57)
	**FP IC**	−12.50 (19.08)	−16.174 (4.155)	−16.58 (3.520)	−17.45 (4.540)	−16.24 (5.625)	−4.57 (25.66)	−15.59 (5.915)	−17.62 (8.255)	−18.723 (7.430)	−18.655 (9.502)
	**FP MEAN**	−12.03 (19.45)	−16.534 (6.033)	−16.90 (4.172)	−17.932 (7.82)	−14.810 (6.020)	−8.522 (24.49)	−18. 216 (7.140)	−18.16 (6.825)	−20.601 (7.591)	−21.562 (9.790)

Legend: ANKLE AND FOOT: AIC = angle of ankle dorsiflexion/plantar flexion at initial contact; AMST = maximum angle of ankle dorsiflexion in stance; AMST = minimum angle of ankle plantar flexion in stance; AMSW = maximum angle of ankle dorsiflexion in swing; A–ROMST = range of motion of ankle in stance; FP IC = foot progression angle at initial contact; FP MEAN = mean value of foot progression. KNEE: KIC = angle of knee flexion at initial contact; KMSW = maximum angle of knee flexion in swing; KMST = minimum angle of knee flexion in stance; K–ROM = range of motion of knee on the sagittal plane; HIP: HIC = angle of hip flexion at initial contact; HMST–MAX= maximum angle of hip flexion/extension in stance; HMST–MIN = minimum angle of hip flexion/extension in stance; HMST–ROM= range of motion of hip flexion/extension in stance; HAA–MAX = maximum angle of hip abduction/adduction; HAA–MIN = minimum angle of hip abduction/adduction; HAA–ROM = range of motion of hip abduction/adduction; HROT–IC = range of motion of hip rotation at initial contact; HROT–MEAN = mean value of hip rotation; PELVIS: PT–IC = angle of pelvic tilt at initial contact; PT–MAX = maximum angle of pelvic tilt; PT–MIN = minimum angle of pelvic tilt; PT–ROM = range of motion of pelvic tilt; PO–MAX = maximum angle of pelvic obliquity; PO min = minimum angle of pelvic obliquity; PO–ROM = range of motion of pelvic obliquity; PR–MAX = maximum angle of pelvic rotation; PR–MIN = minimum angle of pelvic rotation; PR–ROM = range of motion of pelvic rotation; * Statistically significant difference in comparison with baseline (*p* < 0.05). **#** Statistically significant difference in comparison with placebo (*p* < 0.05).

## Data Availability

The datasets used and/or analyzed in the study are available from the corresponding author on reasonable request.
